# Reduced renal function is associated with prolonged hospitalization in frail older patients with non-severe pneumonia

**DOI:** 10.3389/fmed.2022.1013525

**Published:** 2022-09-30

**Authors:** Atsuhiro Kanno, Ryo Kimura, Chika Ooyama, Juri Ueda, Isabelle Miyazawa, Yuko Fujikawa, Shigeru Sato, Nobuo Koinuma, Takahiro Ohara, Kazuhiro Sumitomo, Katsutoshi Furukawa

**Affiliations:** ^1^Division of Community Medicine, Faculty of Medicine, Tohoku Medical and Pharmaceutical University, Sendai, Japan; ^2^Tohoku Medical and Pharmaceutical University, Sendai, Japan; ^3^Miharunomori Clinic, Sendai, Japan; ^4^Morinosato Internal Medicine Clinic, Sendai, Japan; ^5^Department of Internal Medicine, Wakabayashi Hospital, Sendai, Japan

**Keywords:** geriatric medicine, chronic kidney disease, older patient, prolonged hospitalization, frailty, disuse syndrome

## Abstract

**Objective:**

Pneumonia is a disease with high morbidity and mortality among older individuals in Japan. In practice, most older patients with pneumonia are not required ventilatory management and are not necessarily in critical respiratory condition. However, prolonged hospitalization itself is considered to be a serious problem even in these patients with non-critical pneumonia and have negative and critical consequences such as disuse syndrome in older patients. Therefore, it is essential to examine the factors involved in redundant hospital stays for older hospitalized patients with non-severe pneumonia, many of whom are discharged alive.

**Method:**

We examined hospitalized patients diagnosed with pneumonia who were 65 years and older in our facility between February 2017 and March 2020. A longer length of stay (LOS) was defined in cases in which exceeded the 80th percentile of the hospitalization period for all patients was exceeded, and all other cases with a shorter hospitalization were defined as a shorter LOS. In a multivariate logistic regression model, factors determining longer LOSs were analyzed using significant variables in univariate analysis and clinically relevant variables which could interfere with renal function, including fasting period, time to start rehabilitation, estimated glomerular filtration rate (eGFR), the Quick Sequential Organ Failure Assessment (qSOFA) score of 2 or higher, bed-ridden state.

**Results:**

We analyzed 104 eligible participants, and the median age was 86 (interquartile range, 82–91) years. Overall, 31 patients (30.7%) were bed-ridden, and 37 patients (35.6%) were nursing-home residents. Patients with a Clinical Frailty Scale score of 4 or higher, considered clinically frail, accounted for 93.2% of all patients. In multivariate analysis, for a decrease of 5 ml/min/1.73m^2^ in eGFR, the adjusted odds ratios for longer LOSs were 1.22 (95% confidence interval, 1.04–1.44) after adjusting for confounders.

**Conclusion:**

Reduced renal function at admission has a significant impact on prolonged hospital stay among older patients with non-severe pneumonia. Thoughtful consideration should be given to the frail older pneumonia patients with reduced renal function or with chronic kidney disease as a comorbidity at the time of hospitalization to prevent the progression of geriatric syndrome associated with prolonged hospitalization.

## Introduction

Pneumonia was the fifth leading cause of death in 2019, according to the statistical surveys performed by the Ministry of Health Labor and Welfare in Japan (1). The percentage of pneumonia among the causes of death is even more pronounced among individuals older than 65 years of age, resulting in a great impact on the Japanese medical care system currently (1). There is a growing concern that Japan has been progressing into an unprecedented, aged society, and consequently, rising health and nursing care costs for older individuals have become an urgent issue. The problems to be solved are prolonged hospitalization of older individuals and unique circumstances to enforce an extended hospital stay. According to the 2014 statistical survey in Japan, the average length of stay (LOS) in hospital for patients with pneumonia older than 65 years of age was 36.0 days, while that for those older than 75 years of age was 38.4 days (2). Hospitalization periods have important implications for older patients because they may lead to hospitalization-associated functional declines that exacerbates muscle atrophy (3–6), bone loss and cognitive impairment (7). Additionally, these could be described as frailty, a clinical condition of the reduced physiologic reserve, and increased vulnerability to poor health outcomes among older people (8). Especially in patients with less severe pneumonia, it may reduce the quality of life after discharge from the hospital. Although hospitalization may be one of the appropriate and essential treatment options for older patients with pneumonia under some circumstances, long-term hospitalization may contribute to the development of disuse syndrome resulting in delayed discharge.

Chronic kidney disease (CKD) is also considered a serious health threat among older people and is associated with high morbidity and mortality. This is mainly due to the increased prevalence of hypertension, diabetes, and cardiovascular complications, which are risks of CKD in older patients (9). Frailty is also common among CKD patients, even after adjustment for demographics and comorbidities (10). The relationship between frailty and CKD has become a significant public health issue that cannot be overlooked. Additionally, it is reported that patients with CKD had more comorbidities, the highest annual number of inpatients stays, and the greatest number of hospital days compared with other chronic diseases (11), and more extended hospital stays than patients without CKD among infection-related hospitalization (12). However, the association between decreased renal function and prolonged hospitalization has not been adequately studied among older patients under conditions of more common infectious diseases such as pneumonia, especially in situations where the severity of pneumonia itself does not define the length of hospital stay.

In the present study, we analyzed the association between renal function and long-term hospitalization among older patients who admitted to our facility due to non-severe pneumonia and were not needed mechanical ventilation management in order to clarify the factors that shorten the hospital stay.

## Materials and methods

### Study design

The present study was a single-center retrospective observational study. We enrolled patients who were admitted to our facility between February 2017 and March 2020. Patients who had clinical manifestations including fever, cough, shortness of breath, production of sputum, and respiratory crackles on auscultation, in addition to the findings of infiltrates on chest radiography or consolidation on computed tomography, were diagnosed with pneumonia. Patients aged 65 years and older who met the diagnostic criteria of pneumonia on admission were included in the present study. As in-hospital deaths might have caused a bias in the LOS in the hospital, we excluded patients who died during the observation period. Of particular note, there were no cases of COVID-19 reported in Japan during the period of our study, and it is concluded that there were no patients with COVID-19 pneumonia included.

### Participants

A total of 149 patients were eligible for the present study. We excluded patients diagnosed with lung abscesses (*n* = 2), tuberculosis (*n* = 3), drowning (*n* = 1), complicated urinary tract infection (*n* = 6), cellulitis (*n* = 3), and other infections (*n* = 2) on admission and who died due to progression of pneumonia (*n* = 6), in addition to those diagnosed with pneumonia after hospital admission (*n* = 12) and other disorders besides pulmonary disease during hospitalization (*n* = 10). The final analysis was performed with 104 patients, and we adopted only the first event for each patient within the study period. Patients with severe pneumonia are admitted to our hospital in the emergency or respiratory medicine department. Therefore, the elderly pneumonia patients in the present study were limited to older patients who were not indicated for admission to other departments because the degree of pneumonia was not severe enough to require invasive treatment.

### Data collection

We collected data including the baseline demographics, clinical characteristics, comorbid diseases, and social environment before admission, recorded in the medical information forms of the hospitals or primary care facilities from every patient on admission. Each medical history including hypertension, diabetes mellitus, cardiovascular disease, cerebrovascular disease, malignancy, and chronic lung disease was confirmed in the medical records. The prescription of angiotensin converting enzyme inhibitors (ACEI), angiotensin II receptor blockers (ARBs) and diuretics were checked by the letters from previous doctors or medical records, and ACEI and ARBs were described as renin-angiotensin aldosterone inhibitor (RASI) in the present study. We analyzed age as a continuous variable, and age ≥ 85 was defined as advanced age, which was included in the analysis as a categorical variable. The estimated glomerular filtration rate (eGFR) was calculated using the modified Japanese equation (13). The levels of usual activities of daily living (ADLs) before admission obtained through the interviews with family members or caregivers were assessed by skilled physicians and categorized into independent, house-bound, chair-bound, or bed-ridden, according to the categories defined by the Japanese Ministry of Health, Labor and Welfare (14, 15). We used the Clinical Frailty Scale (CFS) to assess frailty among older patients (16). The CFS comprises a functional assessment scale, graded 1–9. Patients with a score >4 requiring help with their usual instrumental ADL are considered clinically frail. The CFS was evaluated by the physician in charge of admission and the nursing staff. Days of fasting and time to start rehabilitation were counted from the date of initiating the fast to that of resumption of oral intake, or the date from admission to the initiation of rehabilitation, respectively. The A-DROP scoring system: (i) Age (male ≥ 70 years, female ≥ 75 years); (ii) Dehydration [blood urea nitrogen (BUN) level ≥ 210 mg/L]; (iii) Respiratory failure (SaO_2_ ≤ 90% or PaO_2_ ≤ 60 mmHg); (iv) Orientation disturbance (confusion); and (v) low blood Pressure (systolic blood pressure ≤ 90 mmHg) proposed by the Japanese Respiratory Society (JRS) was implemented to assess the severity of pneumonia (17). The JRS guidelines recommend that patients with an A-DROP score of 0 would be treated as outpatients, those with a score of 1–2 as outpatients or inpatients depending on the situation, those with a score of 3 as inpatients, and those with a score of 4–5 in an ICU. Therefore, we define pneumonia patients with an A-DROP score of 0–3 as non-severe condition, and those with a score of 4–5 as severe condition. The Quick Sequential Organ Failure Assessment (qSOFA) score has three components: systolic blood pressure < 100 mmHg, respiratory rate ≥ 22 breaths per minute, and altered mental status (Glasgow Coma Scale < 15) (18), and we calculated it based on vital signs on admission to out hospital. Especially, the qSOFA score of ≥ 2 is reported to have strong predictive validity for sepsis (19), and we analyzed and added it in our results.

### Statistical analysis

Continuous variables are shown as medians and interquartile ranges (IQRs: 25–75th percentiles), and categorical variables are shown as percentages. We performed the Shapiro-Wilk test to determine whether continuous variables follow a normal distribution. Statistical differences between medians and categories were compared using the Wilcoxon rank-sum test or the Chi-square test. The primary outcome in the present analysis was the LOS, defined as the days from hospital admission to discharge, and longer LOSs referred to cases in which the 80th percentile of the length of hospitalization among all 104 patients in the final analysis was exceeded. We assessed factors associated with longer LOSs using a univariate logistic regression model. Significant and marginally significant variables in univariate analysis were simultaneously entered into a multivariate logistic regression model. Finally, odds ratios with 95% confidence intervals for longer LOSs were estimated using a logistic regression model. *P-*values < 0.05 were considered significant. We verified the variance inflation factor (VIF) to assess multicollinearity in multiple logistic regression analysis. All analyses were performed using SAS, version 9.4 (SAS Institute, Inc., Cary, NC, USA).

### Ethics statement

This study was approved by the ethical committee of Tohoku Medical and Pharmaceutical University. As the present study is an observational study based on collected clinical data, the study content was disclosed in paper and electronic formats, and each patient could withdraw their consent on the use of their data at any time.

## Results

### Patient characteristics and clinical course

A total of 104 patients admitted to our hospital were enrolled in our study. The baseline clinical characteristics are summarized in Table 1. The median age of all patients at the time of entry was 86 years, and 51.9 % of the patients were female. The dominant comorbidities were hypertension (61.5%) and cardiovascular disease (57.7%). The overall median duration of hospital stay was 26 days in the present study. The percentages of chair-bound and bed-ridden patients in the group from nursing homes were 54.1 and 32.0%, respectively. Most of the patients with pneumonia were classified as mild and moderate in severity according to the A-DROP score. There were no significant differences between the groups with shorter and longer LOSs in baseline clinical characteristics including the severity of frailty [Table 1(a,b)]. Regarding the laboratory findings on admission, the eGFR in the group with shorter LOSs was significantly higher compared to that in the group with longer LOSs [Table 1(c,d)]. The percentage of those with A-DROP ≤ 3 defined as non-severe condition in shorter LOSs were significantly lower than that of those in longer LOSs. Additionally, the percentage of those with qSOFA ≥ 2 in shorter LOSs was higher than that of those in longer LOSs [Table 1(e)]. Between the groups with longer and shorter LOSs, there were no significant differences in admission orders which might lead to disuse syndrome during hospitalization [Table 1(f)], but there were significant differences in the fasting period and duration of antibiotic treatment [Table 1(g)].

**Table 1:** Comparison of basic and characteristics between the two groups with different length of hospitalization; data are shown as *n* (%) or median (interquartile range).

**Variable**	**Shorter LOS**	**Longer LOS**	***P***
	**(*N* = 86)**	**(*N* = 18)**	
(a) Basic characteristics			
Age (years)	87 (82–91)	86 (79–91)	0.827
Female, *n* (%)	46 (53.5)	8 (44.4)	0.485
Weight (kg)	49.0 (38.7–54.5)	44.8 (38.9–47.1)	0.144
Bed-ridden, *n* (%)	51 (61.5)	13 (72.2)	0.389
CFS ≥ 4, *n* (%)	79 (91.9)	18 (100.0)	0.602
Nursing home residents, *n* (%)	29 (33.7)	8 (44.4)	0.388
(b) Comorbidities and Medications			
Hypertension, *n* (%)	57 (66.3)	7 (38.9)	<0.05
Diabetes mellitus, *n* (%)	26 (30.2)	4 (22.2)	0.495
CVD, *n* (%)	48 (55.8)	12 (66.7)	0.397
CeVD, *n* (%)	32 (37.1)	7 (38.9)	0.894
Dementia, *n* (%)	48 (55.8)	9 (50.0)	0.652
Chronic lung disease, *n* (%)	13 (15.1)	5 (27.8)	0.197
Malignancy, *n* (%)	26 (30.2)	3 (16.7)	0.243
Use of RASI, *n* (%)	23 (27.4)	7 (38.9)	0.331
Use of Diuretics, *n* (%)	23 (27.4)	8 (44.4)	0.153
(c) Vital signs			
SBP (mmHg)	141 (122–157)	131 (118–140)	<0.01
DBP (mmHg)	81 (68–93)	77 (70–86)	0.394
HR (beats/min)	87 (76–101)	101 (93–111)	<0.01
GCS < 15, *n* (%)	75 (87.3)	18 (100.0)	0.137
RR (breaths/min)	24 (20–25)	24 (21–25)	0.648
(d) Laboratory findings			
WBC (/μL)	8850 (7030–12650)	10550 (8400–13825)	0.279
Hemoglobin (g/dL)	12.1 (10.9–13.2)	11.9 (11.2–12.7)	0.872
Albumin (g/dL)	3.3 (2.8–3.5)	3.2 (2.7–3.4)	0.243
CRP (mg/dL)	4.56 (1.77–9.99)	5.48 (3.82–9.40)	0.832
BUN (mg/dL)	18 (15–27)	30 (20–39)	<0.05
Creatinine (mg/dL)	0.71 (0.59–0.92)	0.92 (0.70–1.33)	<0.03
eGFR (mL/min/1.73m^2^)	66 (52–82)	47 (31–63)	<0.01
BNP (pg/mL)	83 (43–173)	96 (64–216)	0.352
(e) Severity assessment tools			
A-DROP ≤ 3, *n* (%)	84 (97.7)	15 (83.3)	<0.03
qSOFA ≥ 2, *n* (%)	37 (43.0)	12 (66.7)	0.067
(f) Admission orders			
Bed rest, *n* (%)	50 (61.6)	15 (83.3)	0.102
Dental intervention, *n* (%)	23 (26.7)	9 (50.0)	0.083
Placement of urinary catheter, *n* (%)	52 (60.5)	14 (77.8)	0.191
Rehabilitation, *n* (%)	71 (82.6)	17 (94.4)	0.295
(g) Clinical course			
Fasting period (days)	4 (1–7)	7 (5–8)	<0.01
Duration of Rehabilitation (days)	21 (14–29)	52 (45–72)	<0.001
Duration of antibiotic treatment, (days)	8 (6-12)	19 (14–22)	<0.001
Length of hospitalization (days)	21 (16–30)	56 (51–78)	<0.001
Transfer to another facility, *n* (%)	33 (38.4)	14 (77.8)	<0.001

LOS, length of stay; CVD, cardiovascular disease; CeVD, cerebrovascular disease, RASI, renin-angiotensin-aldosterone system inhibitor; SBP, systolic blood pressure; DBP, diastolic blood pressure; HR, heart rate; GCS, Glasgow Coma Scale; RR, respiratory rate; WBC, white blood cell; CRP, C-reactive protein; BUN, blood urea nitrogen; eGFR, estimated glomerular filtration rate; BNP, brain natriuretic peptide; qSOFA, Quick Sequential Organ Failure Assessment.

### Factor determining LOS and predictors of long-term hospitalization

The univariate analysis revealed the “fasting period,” “time to start rehabilitation, and eGFR were significantly correlated with longer LOSs (Table 2). In addition, the multivariate logistic analysis including clinical parameters as stated in Table 3 revealed that eGFR, time to start rehabilitation and bedridden state were significantly correlated with longer LOSs. Additionally, we examined the association between eGFR classification based on CKD grade and outcome, but no significant difference was found when adjusted for confounding factors. Furthermore, the VIF was consistently < 10 for the clinical parameters, and this indicated no multicollinearity among the independent variables.

**Table 2:** Analysis of factors associated with longer LOSs in univariate analysis.

	**OR**	**95% CI**	***P***
Bedridden state	2.77	0.98–7.88	0.056
Fasting period (day)	1.05	1.01–1.09	<0.001
Time to start rehabilitation (day)	1.26	1.03–1.54	<0.03
Indwelling urinary catheter	2.29	0.69 – 7.54	0.174
qSOFA score ≥ 2	2.65	0.91–7.71	0.074
Age (year)	0.99	0.93–1.08	0.955
Advanced age (≥ 85 years)	1.03	0.36 – 2.91	0.959
Female	0.69	0.25–1.93	0.486
eGFR* (−5ml/min/1.73m^2^)	1.17	1.04–1.32	<0.01
Albumin (g/dl)	0.54	0.22–1.31	0.174
Hemoglobin (g/dl)	1.01	0.78–1.29	0.994
CRP (mg/dL)	1.03	0.95–1.11	0.469
Use of RASI	1.69	0.58–4.88	0.334
Use of diuretics	2.12	0.75–6.04	0.159
Hypertension	0.99	0.91–1.10	0.988
Diabetes mellitus	0.66	0.19–2.19	0.498
Chronic pulmonary disease	2.16	0.66–7.09	0.204
Cardiovascular disease	1.58	0.54–4.61	0.399
Cerebrovascular disease	1.07	0.38–3.05	0.894

LOS, length of stay; OR, odds ratio; CI, confidence interval; eGFR, estimated glomerular filtration rate; qSOFA, quick sequential organ failure assessment; RASI, renin-angiotensin-aldosterone system inhibitor.

* Every 5-estimated glomerular filtration rate on admission decrement.

**Table 3:** Analysis of factors associated with longer LOSs in multivariate analysis.

	**OR**	**95% CI**	***P***
Bedridden state	6.01	1.24–29.17	0.026
Fasting period (day)	1.04	0.98–1.10	0.134
Time to start rehabilitation (day)	1.29	1.03–1.60	0.024
eGFR* (−5ml/min/1.73m^2^)	1.22	1.04–1.44	0.017
qSOFA score ≥ 2	0.27	0.06–1.25	0.093

LOS, length of stay; OR, odds ratio; CI, confidence interval; eGFR, estimated glomerular filtration rate; qSOFA, quick sequential organ failure assessment.

* Every 5-estimated glomerular filtration rate on admission decrement.

## Discussion

Herein, we analyzed factors associated with longer LOSs among older patients with non-severe pneumonia in a single-center retrospective observational study. For older patients with non-critical pneumonia who could be managed in a general medical floor not in the ICU, it was concluded that reduced renal function was significantly associated with longer hospitalization irrespective of confounders including the use of drugs that may affect renal function, qSOFA score at a level suggestive of sepsis in addition to factors to accelerate the geriatric syndrome among older inpatients. Few reports have studied factors associated with length of hospital stay in non-serious older pneumonia, therefore, it is important to examine the factors associated with long-term hospitalization in this population.

Previous studies have revealed several factors associated with LOSs in older patients with pneumonia as follows: advanced age (20, 21), functional status (20), diabetes mellitus (21), chronic pulmonary disease (21), the pneumonia severity index (21), hypoalbuminemia (21, 22), dysphagia (on tube feeding) (22), chronic kidney disease (CKD) (22), indwelling urethral catheter implementation (23). While our results support a part of these factors, the previous results also remind us that there are the specific problems affect long LOSs among the older patients, such as multimorbidity and geriatric syndrome.

Prolonged LOSs are considered to lead to the development and progression of frailty in older individuals via further deterioration of functional status (3–5). Deteriorated function would require greater time to restore patients' ADLs to the pre-hospitalization levels, and, moreover, the progression of the disuse syndrome could be difficult to be restored with the extension of hospitalization period. Added to treatment of pneumonia itself, it is also necessary to consider the factors that contribute to prolonged hospitalization in order to prevent the disuse syndrome for older patients. Therefore, in the initial stage of hospitalization it is essential to identify factors that may affect LOSs among older patients with pneumonia.

In our study, reduced eGFRs were significantly correlated with the incidence of longer LOSs. Interestingly, reduced eGFRs were demonstrated to be associated with a remarkably increased risk of infection from the early stage of CKD (24, 25). Furthermore, reduced eGFR was reported to be associated with increased risk of hospitalization with pneumonia, and a graded association between the progression of CKD and risk of hospitalization with pneumonia has been reported (26). The pathological mechanisms of CKD may be partially explained as impairment of the immune system, which includes impaired leukocyte and neutrophil functions and reduced lymphocyte production (27–29). Additionally, another study reported the increased level of inflammatory marker among patient with CKD and revealed that there was an inverse relationship between plasma levels of inflammatory cytokines and eGFR (30, 31). These studies support the assumption that impaired immune system and inflammation may contribute a potential susceptibility to infection and delayed recovery from infectious disease among patients with CKD.

It has previously been reported that CKD is associated with prolonged LOSs (22), however, the present study differs from previous study in the following important aspects. First, the present report is based solely on older subjects, while previous study consisted of heterogenous populations, including younger subjects. Second, previous study has analyzed CKD as a comorbidity, they have not employed and evaluated eGFR as a continuous variable as in the present study. Therefore, the precise relationship with renal function was not evaluated in the previous study. Third, the previous study has included a large number of subjects with very severe pneumonia in the aged patients. This is a possibility that prolonged LOSs was strongly influenced by delayed recovery from severe acute pneumonia, frailty as an underlying condition, low nutrition, hypertension, and diabetes as comorbidities in older patients. These mean that there exists a difference in factors related to prolonged LOSs depending on the pneumonia condition of the subject patients.

On the other hand, CKD and frailty are closely related to CKD (10). In our present study, the two groups had very high rates of clinical frailty, and although there was no difference, the effect on prolonged LOSs might have been strong. Although frailty may be mild to moderate, it is reasonable to assume the invasiveness of pneumonia and the reduced resilience of the elderly. Additionally, the worsening levels of medically-induced sarcopenia and frailty associated with rest and fasting during hospitalization all contributed to the delayed recovery of physical fitness.

In the present study, reduced eGFR has a significant impact on prolonged hospital stay among older patients with non-critical pneumonia after considering the factors that affect the duration of hospitalization. It is also suggested that the potentially impaired immunity in frail older patients with decreased renal function may have contributed to delayed healing of pneumonia. However, in relation to the assessment of baseline renal function, there were no sufficient patient information in the hospital administrative data based on a reference letter from their previous doctors in our study. Therefore, we could not correctly categorize patients with the reduced renal function into AKI, CKD or acute exacerbation of CKD.

The strength of our study is that we could effectively evaluate the precise relationship between factors other than the condition of the disease itself and LOS because our participants are non-severe pneumonia patients and discharge as a disposition is a more realistic issue for them. There were some limitations to the present study. First, our analyses did not include clinical and biological information, such as causative organisms, previous vaccinations, and the selection of antibiotics that might have affected the treatment. Second, the definition of LOSs has not yet been established and is still diverse depending on previous studies (32). Third, the present study participants contain a small sample size from the perspective of events per variable required for a logistic regression analysis. There is a possibility that this may affect the result. Finally, there is selection bias, because only patients with mild or moderate pneumonia were recruited in our study. Patients with serious systemic conditions requiring endotracheal intubation and management in the intensive care unit were excluded because they were treated at the emergency department.

## Conclusion

We demonstrated that reduced kidney function at admission is associated with the length of hospitalization among older patients with non-severe pneumonia in addition to the factors previously considered to exacerbate the geriatric syndrome. It is suggested that thoughtful consideration should be provided to patients with pneumonia with reduced eGFRs and a detailed study is needed to determine if this could be related to other in-hospital outcomes.

## Data availability statement

The raw data supporting the conclusions of this article will be made available by the authors, without undue reservation.

## Ethics statement

The studies involving human participants were reviewed and approved by the Ethical Committee of Tohoku Medical and Pharmaceutical University. Written informed consent for participation was not required for this study in accordance with the national legislation and the institutional requirements.

## Author contributions

AK contributed to conception and design of the study. AK, RK, JU, IM, CO, YF, SS, NK, TO, and KS acquired and organized the database. AK and RK performed analysis and interpretation of data. AK and KF wrote the first draft of the manuscript. KF led critical revision of the manuscript for important intellectual content. All authors contributed to manuscript revision, read, and approved the submitted version.

## Conflict of interest

The authors declare that the research was conducted in the absence of any commercial or financial relationships that could be construed as a potential conflict of interest.

## Publisher's note

All claims expressed in this article are solely those of the authors and do not necessarily represent those of their affiliated organizations, or those of the publisher, the editors and the reviewers. Any product that may be evaluated in this article, or claim that may be made by its manufacturer, is not guaranteed or endorsed by the publisher.
